# Dietary Patterns and Their Association with Cardiometabolic Biomarkers and Outcomes among Hispanic Adults: A Cross-Sectional Study from the National Health and Nutrition Examination Survey (2013–2018)

**DOI:** 10.3390/nu15214641

**Published:** 2023-11-01

**Authors:** Brandon Osborn, Matthew A. Haemer

**Affiliations:** Section of Nutrition, Department of Pediatrics, University of Colorado Anschutz Medical Campus, 12700 E 19th Ave F561, Aurora, CO 80045, USA

**Keywords:** dietary pattern, Hispanic adults, cardiometabolic outcomes, NHANES

## Abstract

Cardiovascular disease and metabolic disorders are disproportionately prevalent among Hispanic and Latino adults in the United States. We extracted a posteriori dietary patterns (DPs) among a nationally representative sample of 2049 Hispanic adults using the 2013–2018 National Health and Nutrition Examination Survey. Three primary DPs and their tertiles were identified, and their associations with cardiometabolic outcomes were examined. Those with higher levels of the Solids Fats, Cheeses, Refined Carbohydrates DP were more likely younger, male, and Mexican American. Those with higher levels of the Vegetables DP were more likely female, higher income, and long-term immigrant residents. Those with higher levels of The Plant-Based DP tended to have higher education levels. Higher levels of the Solid Fats, Cheeses, Refined Carbohydrates DP level were positively associated with body mass index (Tertile 2, β: 1.07 [95%CI: 0.14, 1.99]) and negatively associated with lower high-density lipoprotein cholesterol (HDL-C) levels (Tertile 3, β: −4.53 [95%CI: −7.03, −2.03]). Higher levels of adherence to the Vegetables DP were negatively associated with body fat (Tertile 3, β: −1.57 [95%CI: −2.74, −0.39]) but also HDL-C (Tertile 2, β: −2.62 [95%CI: −4.79, −0.47]). The Plant-Based DP showed no associations with cardiometabolic outcomes. Future research and interventions should consider these associations as well as the sociodemographic differences within each DP.

## 1. Introduction

Cardiovascular disease (CVD) and metabolic disorders, collectively referred to as cardiometabolic outcomes, pose a significant health burden. Among the various populations affected, Hispanic and Latino adults in the United States have shown a disproportionate prevalence of these conditions, often characterized by a higher prevalence of obesity and diabetes. For example, Hispanic adults are more likely to have type 2 diabetes (17%) compared to non-Hispanic White adults (8%) [1]. Hispanic adults are also more likely to have age-adjusted obesity (45.6%) compared to non-Hispanic White adults (41.4%) [2]. The intricate interplay between genetic predisposition, the disparate impacts of social determinants, cultural practices, and dietary choices underscores the need for a deeper understanding of dietary patterns in this population and their potential impact on cardiometabolic health.

Diet plays a pivotal role in the development and progression of cardiometabolic diseases. Evidence suggests that dietary patterns, encompassing the overall composition and combination of foods consumed, may have a more comprehensive influence on health outcomes than individual nutrients or specific food items [3]. However, the majority of research examining dietary patterns utilizing data-driven methods has been conducted on predominantly non-Hispanic populations, limiting the generalizability of findings to the unique sociocultural and dietary contexts of Hispanic communities [4]. Consequently, there exists a critical gap in the literature regarding the associations between dietary patterns and cardiometabolic outcomes, specifically among Hispanic adults. Of the studies examining diet and cardiometabolic outcomes among Hispanics, dietary data usually comprise indices, are sourced from non-nationally representative samples, and usually focus on individual disease-oriented outcomes such as obesity, diabetes, or cardiovascular disease [5,6].

This manuscript aims to address this research gap by presenting a detailed investigation into the dietary patterns of Hispanic adults in the United States and their associations with cardiometabolic outcomes. By utilizing nationally representative data, this study seeks to provide a comprehensive analysis that captures the diversity within the Hispanic population while also accounting for the complex interplay between sociodemographic factors. The utilization of nationally representative data enables us to explore a wide array of dietary patterns among Hispanic adults, including variations based on nativity and duration, socioeconomic status, and other factors. We address the following research questions in this paper: (1) Which dietary patterns are the most prominent among a nationally representative sample of Hispanic adults in the United States? (2) What are the sociodemographic characteristics within tertiles of each of these dietary patterns? (3) Are the tertiles of these dietary patterns associated with cardiometabolic biomarkers and outcomes?

## 2. Materials and Methods

### 2.1. Study Population

We used data from the National Health and Nutritional Examination Survey (NHANES) [7] from 2013 to 2014, 2015 to 2016, and 2017 to 2018. The NHANES is a publicly available data set provided by the National Center for Health Statistics, a division of the Centers for Disease Control and Prevention. It is designed to assess both the health and nutritional status of adults and children in the United States [8]. The NHANES consists of a series of interviews, physical examinations, and laboratory measurements conducted in relation to about 5000 persons each year, which is representative of the noninstitutionalized population in the United States. The sample was selected using a complex, stratified, multistage probability cluster design. Participants in this study completed the family, household, and dietary questionnaires, which included demographic, socioeconomic, dietary, and health-related questions [9]. Participants in this study also participated in the examination component at a Mobile Examination Center (MEC); this component consisted of medical, dental, and physiological measurements as well as laboratory tests administered by highly trained medical personnel [9,10].

### 2.2. Analytic Sample and Weighting

We restricted our analytic sample to adults aged 18 and older and those who identified as “Mexican American” or “other Hispanic” when asked about their race and ethnicity (n = 4585). We further restricted inclusion to those participants who had completed two separate reliable 24-h dietary recalls (n = 2376). This decline in sample size was due to the lower proportion of Hispanic adults who had undergone two 24-h dietary recalls. Reliable dietary recalls must meet certain criteria as defined by the NHANES, which include assessing the quality and completeness of a survey participant’s response to the dietary recall section. Finally, we further restricted inclusion to those participants who had data for all demographic questions on age, sex, income, level of education, and nativity and duration of residence in the United States, resulting in a total sample size of 2049 participants. NHANES provides guidelines for creating sample and variance units for public-use data files, including sample weights for selected subsamples such as the fasting subsample. We used the appropriate sampling weights in our statistical analyses to provide reasonable, unbiased, and design-consistent estimates of variance [11,12]. Based on NHANES guidelines, two-year sample weights from the 2013–2014, 2015–2016, and 2017–2018 waves were combined by dividing the two-year weights for each cycle by three to produce 6-year estimates [11].

### 2.3. Dietary Patterns

Dietary intake was assessed in the NHANES study via two separate dietary recalls. All NHANES participants are eligible for two 24-h dietary recalls. The first dietary recall is collected in person at the MEC, and the second recall, which only a subset of participants complete, is collected via telephone 3 to 10 days later. Both recalls are offered in English and Spanish as well as other languages [10]. Dietary intake is first weighted across the two dietary recall days in order to provide more precise estimates of consumed portions [13]; then, it is disaggregated into ingredients using the Nutrient Database for Dietary Studies, which provides nutrient compositions for about 8700 foods reported in What We Eat In America, which are the dietary intake components of the NHANES [14]. Dietary data are then converted into the respective amounts of food pattern equivalents present in them [15] and assigned to a United States Department of Agriculture food pattern component listed in the Food Patterns Equivalent Database (FPED). The food pattern components are measured as cup equivalents of fruit, vegetables, and dairy; ounce equivalents of grains and protein foods; teaspoon equivalents of added sugars; gram equivalents of solid fats and oils; and the number of alcoholic drinks consumed [16]. This conversion process results in a total of 37 FPED food groups or components.

For this study, we identified and used mutually exclusive food groups (28 FPED components). We excluded food groups that in total or in aggregate consisted of two or more food groups. The excluded duplicative, total, or aggregate groups were beans and peas (computed as protein foods); total fruits; total vegetables; total red and orange vegetables; total starchy vegetables; total grains; total protein foods; total meat, poultry, and seafood; and total dairy. The 28 FPED components used in this study were citrus, melons, and berries; other fruits; fruit juice; dark-green vegetables; tomatoes; other red and orange vegetables; potatoes; other starchy vegetables; other vegetables; beans and peas (computed as vegetables); whole grains; refined grains; meat; cured meat; organ meat; poultry; seafood high in omega-3 fatty acids; seafood low in omega-3 fatty acids; eggs; soy products; nuts and seeds; milk; yogurt; cheese; oils; solid fats; added sugars; and alcoholic drinks.

We then used exploratory factor analysis, a data reduction technique, to identify unique dietary patterns within the 28 food groups [17]. This method reduced the food groups (i.e., indicators) to a few mutually exclusive patterns (i.e., factors) while minimizing the loss of information [17]. Prior to conducting principal component analysis, we generated a correlation matrix to identify the correlations among the 28 food groups. We evaluated the appropriateness of the data for factor analysis based on Kaiser–Meyer–Olkin (KMO) and Bartlett’s Test of Sphericity (homogeneity of variance) values. The KMO measure, which represents the adequacy of sample size, compares the value of partial correlation coefficients against the total correlation coefficients.

We then used principal component analysis (PCA) to identify unique dietary factors from the 28 food groups. We conducted orthogonal rotation with the varimax option to derive non-correlated factors and minimize the number of indicators (i.e., food groups) that had high loading on one factor [18,19]. The first factor extracted is the one that accounts for the greatest variance in the dataset, while the second factor, independent of the first, explains the largest possible share of the remaining variance, and so on, without the components being correlated with each other. Three selection criteria were used to determine which factors should be retained and later included in regression models: (1) factors with an eigenvalue greater than one, (2) interpretation of a scree plot, and (3) the interpretable variance percentage [17]. The principal components, or factors, are unique dietary patterns that are named primarily based on the food groups that loaded highly within each factor (Table S1). We then calculated demographic and socioeconomic characteristics for participants across tertiles of each dietary pattern. We tested for differences across tertiles using Chi-Square tests for categorical variables and adjusted Wald Tests for continuous variables (Table 1, Table 2 and Table 3).

Once we selected which factors to retain, we computed predicted scores of every retained factor for each respondent using the *predict* command in Stata. These scores were subsequently utilized for both data exploration and prediction models [20]. These predicted factor scores were derived by multiplying each food group’s factor loading by a respondent’s corresponding food group value (scoring coefficient) and then summing them across food groups to determine a participant’s factor score for each dietary pattern. We then standardized these predicted scores (mean scaled to 0, and standard deviation scaled to 1) in order to make the results comparable across all retained dietary patterns. These standardized predicted dietary pattern scores became the independent variable in subsequent regression models.

### 2.4. Dependent Variables

Our dependent variables consisted of laboratory, clinical, and self-report measures related to cardiometabolic health that are available in the NHANES. We examined body mass index, type 2 diabetes (0 = No, 1 = Yes), glycohemoglobin levels, body fat percentage obtained via dual-energy x-ray absorptiometry (DXA), self-reported history of myocardial infarction (0 = No, 1 = Yes), self-reported history of coronary heart disease (0 = No, 1 = Yes), high-density lipoprotein cholesterol levels (mg/dL), fasted low-density lipoprotein Cholesterol levels (mg/dL), fasted triglycerides (mg/dL), systolic blood pressure (mmHg), and diastolic blood pressure (mmHg). Additional information about these measures can be obtained on the NHANES website [10].

### 2.5. Covariates

*Covariates* for all models included age (years); sex (male, female); education level (less than high school diploma, high school diploma or equivalent, some college or technical training, and university graduate or greater); income (USD 0–19,999, USD 20,000–34,999, and USD 35,000+); and caloric intake (average intake of kcals from two 24-h dietary recalls). These demographic variables are highly correlated with dietary intake and cardiometabolic outcomes. We controlled for caloric intake as we wanted to test the associations between dietary patterns and cardiometabolic outcomes independent of energy. For type 2 diabetes and glycohemoglobin, we also controlled for whether participants were on a special diet tailored for diabetes (yes/no), currently taking insulin (yes/no), or currently taking medication to control blood sugar levels (yes/no). For history of coronary heart disease and myocardial infarction, we also controlled for smoking. For high-density-lipoprotein cholesterol, we also controlled for physical activity levels (METS minutes per week). For triglycerides, we also controlled for glycohemoglobin levels. For both systolic and diastolic blood pressure levels, we also controlled for smoking.

### 2.6. Multivariable Regression

We examined the association between the tertiles of the dietary patterns and cardiometabolic outcomes. While our aforementioned selection criteria identified 11 unique factors, we only retained three different dietary patterns as outcomes in the regression models, as the first three factors explained the most variance and had the most interpretable dietary patterns. We regressed scores for each of the three unique dietary patterns on our cardiometabolic outcomes using linear regression for continuous variables and logistic regression for binary variables. We included the following covariates in all regression models: age, sex, education, income, and caloric intake. We conducted sensitivity analyses by including additional covariates (smoking, insulin use, physical activity, HbA1c, special diet tailored for diabetes, and current use of medication to control blood sugar) through a stepwise regression approach in all models, but not all models were impacted by the addition of these covariates. All statistical analyses were conducted using Stata 16 IC, StataCorp LLC, College Station, TX, USA, [21].

## 3. Results

### 3.1. Dietary Patterns and Sociodemographic Characteristics

There was a sufficient degree of correlation between the different foods to allow us to proceed with factor analysis based on the value of the KMO test (0.69). The results of the Bartlett test of sphericity were highly significant (*p* < 0.001), indicating homogeneity of variance through the consumption of foods. Eleven components, or factors, were extracted via factor analysis for different food groups using the criteria stated above. The 11 factors had an eigenvalue over 1, accounting for 58.4% of the total observed variation in the food-consumption patterns among Hispanics (Table S1). Only food groups with absolute value loadings greater than or equal to 0.35 [22] were retained and considered significant contributors to each factor (Table S1).

The rest of the results focus on the first three factors, since these factors were the only ones included in regression models. These three factors accounted for 25.8% of the total observed variation in the food consumption patterns among Hispanics. The first factor, which accounted for 11.9% of the total variance, was labeled as the Solid Fats, Cheeses, and Refined Carbohydrates dietary pattern, as high factor loadings (0.35 or greater) were observed for solid fats, refined grains, cheese, added sugars, and tomatoes and tomato products. The second factor, which accounted for 8.7% of the total variance, was labeled as the Vegetables dietary pattern, as high factor loadings were observed for red and orange vegetables, tomato and tomato products, dark-green vegetables, and other vegetables. The third factor, which accounted for 5.2% of the total variance, was labeled as the *Plant-based* dietary pattern, as high factor loadings were observed for soy, nuts and seeds, whole fruits, and red and orange vegetables.

We examined the sample characteristics for each of the three dietary patterns and found significant differences across the tertiles of each dietary pattern score corresponding to specific demographic and socioeconomic variables (Table 1, Table 2 and Table 3). For the Solid Fats, Cheese, and Refined Carbohydrates dietary pattern (Table 1), there were significant differences in terms of age. On average, people who scored highly with respect to this pattern were younger than those who had low scores (highest tertile: 37.9 years; lowest tertile: 42.8 years). There were also sex differences such that men were more likely to score higher on this pattern compared to women (highest tertile: 60.8% male; lowest tertile: 32.4% male). Mexican Americans made up a higher proportion of this dietary pattern compared to other Hispanics, and they made up 67.4% of the highest tertile, which can be compared to 54.3% in the lowest tertile. There were no significant differences in terms of education level or nativity and duration of residence across the tertiles.

For the Vegetables dietary pattern (Table 2), there were significant sex differences such that women were more likely to be in the median tertile (56.2%) compared to men (43.8%). There were also significant differences in terms of household income levels. In the highest tertile, 15.3% of the respondents had household incomes less than USD 20,000, whereas, in the lowest tertile, 30.1% of the respondents had household incomes less than USD 20,000, indicating that those who scored lower on this pattern were more likely to live in a low-income household. Lastly, there were significant differences with respect to nativity and duration of residence. US-born respondents were less likely to score highly on this pattern compared to immigrants living in the US for 10 years or more. There were no significant differences with respect to education level or Hispanic origin across the tertiles.

For the Plant-Based dietary pattern (Table 3), the only significant difference across tertiles was in terms of education level, whereas in the highest tertile, 51.0% of the respondents had some level of higher education, and in the lowest tertile, 43.6% of the respondents had some level of higher education. This result indicates that those who scored higher on this pattern were more likely to have a higher level of education.

### 3.2. Regression Models

#### 3.2.1. Solid Fats, Cheeses, and Refined Carbohydrates Dietary Pattern

The results of the regression models are depicted in Table 4, Table 5 and Table 6. For the Solid Fats, Cheeses, and Refined Carbohydrates dietary pattern, there were statistically significant associations with body mass index (BMI) and high-density lipoprotein cholesterol. Persons in the second and highest tertiles had higher BMIs than those in the lowest tertile, with those in the second tertile having a statistically significant higher BMI. Persons in the second and highest tertiles had a lower high-density lipoprotein cholesterol (HDL-C) value than those in the lowest tertile, with those in the highest tertile having a statistically significant lower value. There were no statistically significant associations between the Solid Fats, Cheeses, and Refined Carbohydrates dietary pattern and other cardiometabolic outcomes.

#### 3.2.2. Vegetables Dietary Pattern

For the Vegetables dietary pattern, there were statistically significant associations with body fat percentage and HDL-C. Persons in the second and highest tertiles had lower body fat levels than those in the lowest tertile, with those in the highest tertile having statistically significant lower levels of body fat. Persons in the second tertile had a statistically significant lower HDL-C value than those in the lowest tertile. However, while not statistically significant, persons in the highest tertile had a higher HDL-C value than those in the lowest tertile. There were no statistically significant associations between the Vegetables dietary pattern and other cardiometabolic outcomes.

#### 3.2.3. Plant-Based Dietary Pattern

There were no statistically significant associations between the Plant-Based dietary pattern and cardiometabolic outcomes.

## 4. Discussion

We extracted and identified three unique dietary patterns that were the most common among a nationally representative sample of Hispanic adults. We then examined the sociodemographic characteristics within the tertiles of each dietary pattern as well as the association between the tertiles of the dietary patterns and eleven cardiometabolic outcomes. We found that higher levels of adherence to the Solid Fats, Cheeses, and Refined Carbohydrates dietary pattern were positively associated with body mass index and negatively associated with high-density lipoprotein cholesterol. Higher levels of adherence to the Vegetables dietary pattern were negatively associated with body fat percentage but also with high-density lipoprotein cholesterol. We found no associations between the Plant-Based dietary pattern and cardiometabolic outcomes.

### 4.1. Diverse Dietary Patterns and Demographic Variations

This study’s results reveal significant associations between dietary patterns and various demographic and socioeconomic factors, providing a deeper understanding of the complex relationship between diet and cardiometabolic health among a nationally representative sample of Hispanic adults living in the United States.

For the Solid Fats, Cheese, and Refined Carbohydrates dietary pattern, it is noteworthy that individuals with higher scores tended to be younger, male, and more likely to be of Mexican American origin. These findings suggest that younger Hispanic adults, particularly males of Mexican American descent, are more prone to consuming diets characterized by high amounts of solid fat, cheese, and refined carbohydrates. This demographic variation highlights the importance of targeted interventions for specific subgroups within the Hispanic population.

Conversely, the Vegetables dietary pattern was associated with differences in sex, income, and nativity/duration of residence. Women were more likely to adhere to this pattern, as were individuals with higher household incomes, indicating that women and those with a high-income status prioritize vegetable consumption in their diets. Additionally, immigrants living in the US for 10 years or more were more likely to follow this pattern compared to US-born Hispanics, potentially demonstrating protective factors attributed to this population that may be related to health selection characteristics (the immigration of healthier individuals to the United States) as well as selective emigration (i.e., the migration of less-healthy individuals back to their home countries) [23] and less acculturation, specifically less dietary acculturation [24,25,26,27].

The Plant-Based dietary pattern was associated with higher levels of education, with individuals who had attained higher education being more likely to follow this pattern. This finding aligns with previous research that has shown a positive correlation between educational attainment and the adoption of plant-based diets, reflecting a greater awareness of the health benefits associated with these dietary choices [28,29].

### 4.2. Associations with Cardiometabolic Outcomes

The regression models provided insights into the associations between dietary patterns and cardiometabolic outcomes among Hispanic adults. For the Solid Fats, Cheese, and Refined Carbohydrates dietary pattern, higher adherence was associated with higher body mass index (BMI) values and lower levels of high-density lipoprotein cholesterol (HDL-C). These results are consistent with the established understanding that diets rich in solid fats and refined carbohydrates contribute to obesity and unfavorable lipid profiles [30]. These associations highlight the significance of addressing this particular dietary pattern in interventions intended to reduce cardiometabolic risk among Hispanic adults, especially younger males of Mexican American origin who are more likely to follow this pattern.

In contrast, the Vegetables dietary pattern was associated with lower body fat percentages, constituting a positive health outcome, indicating that individuals adhering to this pattern tend to have healthier body compositions. However, it is important to note that there was a statistically significant lower HDL-C value among individuals in the second tertile of adherence, although those in the highest tertile exhibited a higher, albeit non-statistically significant, HDL-C value compared to those in the lowest tertile. These findings suggest a complex relationship between vegetable consumption and HDL-C levels that warrants further investigation.

Surprisingly, the Plant-Based dietary pattern did not demonstrate statistically significant associations with any of the cardiometabolic outcomes assessed in this study. This result could be attributed to the diverse nature of this dietary pattern, as it encompassed a wide range of foods with varying nutrient compositions, including red vegetables, soy, nuts and seeds, and fruits. The individual choices within this dietary pattern may have different effects on health, and further research is needed to explore these variations.

### 4.3. Limitations and Strengths

This study had both limitations and strengths. Using the nationwide NHANES data from 2013–2018 was a strength because it included a large, diverse sample of Hispanic adults. In addition, the use of a posteriori derived dietary patterns, rather than a priori indices, was a strength, as they highlight actual dietary patterns that a representative sample of Hispanic adults, in aggregate, adhered to in the United States from 2013 to 2018. Another strength is our use of the average of dietary recalls rather than relying on just one 24-h dietary recall, with the latter being less likely to reflect usual intake. Key limitations of this study include the cross-sectional nature of the data and study design, which prevented us from inferring causality or directionality. Additionally, we reported our logistic regression results utilizing adjusted odds ratios for three dichotomous outcomes (diabetes status, history of coronary heart disease, and history of myocardial infarction). Odds ratios might have led to overestimation of the associations among these outcomes since we utilized cross-sectional data in our study and thus introduced potential bias. However, none of these associations were statistically significant. We chose to utilize odds ratios, rather than prevalence ratios or predicted probabilities, in order to increase the interpretability of comparing the associations within (tertiles) and between dietary patterns among the 11 cardiometabolic outcomes. An additional limitation is that the dietary data and some of the cardiometabolic measures relied on self-report data, including respondents’ history of coronary heart disease and myocardial infarction. Self-report measures are subject to both recall and social desirability biases [22,31]. If specific dietary intake is underreported, some of these associations may be stronger or weaker depending on which food groups are underreported. Additionally, the two outcomes reliant on self-report responses concerning whether a participant had ever been told by a physician that they had coronary heart disease or myocardial infarction might have resulted in underreporting among respondents that may have limited access to healthcare. Future studies should consider these limitations and utilize longitudinal designs as well as history of medical events such as myocardial infarction derived from electronic health records.

### 4.4. Implications and Future Directions

The findings from this study offer valuable insights into dietary patterns and their associations with cardiometabolic outcomes among a nationally representative sample of Hispanic adults. They emphasize the importance of considering demographic, socioeconomic, and cultural factors when designing interventions and public health strategies intended to improve cardiometabolic health within this population. Targeted interventions should be developed to address specific dietary patterns associated with unfavorable cardiometabolic outcomes, such as the Solid Fats, Cheese, and Refined Carbohydrates dietary pattern. These interventions should be tailored to the unique needs of subgroups within the Hispanic population, taking into account age, gender, ethnicity, income, and acculturation status. For example, we found that younger Hispanic adults, particularly males of Mexican American descent, are more prone to consuming diets characterized by high levels of solid fat, cheese, and refined carbohydrates. This highlights the importance of targeting and tailoring interventions for specific demographic subgroups within the broader Hispanic population.

Future researchers should explore the mechanisms underlying these associations, investigate the impact of specific dietary components on metabolic pathways, and conduct longitudinal studies to establish causality. Additionally, comparing dietary patterns and their associations with cardiometabolic outcomes between various groups in the United States will help provide more insight into personalized nutrition. Future studies examining dietary patterns and cardiometabolic outcomes among Hispanics and Latinos should examine differences by subgroup as there is much diversity within these aggregated groups. Additionally, qualitative research methods could be employed to gain deeper insights into the cultural and social factors, as well as the preferences and traditions, that influence dietary choices among Hispanic adults.

## 5. Conclusions

In conclusion, this study’s results provide critical information for policymakers, healthcare providers, and researchers seeking to address health disparities and promote cardiometabolic health among Hispanic communities in the United States. By understanding the complex interplay between dietary patterns, sociodemographic factors, and health outcomes, we can develop more effective strategies to reduce the burden of cardiometabolic diseases within this population and advance health equity.

## Figures and Tables

**Table 1:** Sample characteristics of Hispanic adult respondents within the Solid Fats, Cheeses, and Refined Carbohydrates Dietary Pattern included in the National Health and Nutrition Examination Survey, 2013–2018 (n = 2049).

Characteristics		Tertiles			
	Overall	T1	T2	T3	
N	2049	683	683	683	
Age, Mean (In Years)	40.2	42.8	41.0	37.9	
Sex, %					
Female	51.7	67.6	55.2	39.2	***
Male	48.3	32.4	44.8	60.8	***
Household Annual Income, %					
USD 0–19,999	20.8	22.5	21.7	19.1	**
USD 20,000–34,999	21.4	28.3	19.1	18.8	**
USD 35,000+	57.8	49.2	59.2	62.1	**
Education Level, %					
Less than High School Education	34.6	38.5	28.6	36.8	*
High School Diploma	21.9	21.4	23.4	21.1	*
Some College+	43.4	40.1	48.0	42.1	*
Nativity and Duration of Residence, %					
US-Born	49.1	46.1	50.5	50.1	
Living In the US <10 Years	10.6	11.0	9.7	10.9	
Living in the US for 10 Years+	40.3	43.0	39.8	39.0	
Race/Hispanic Origin, %					
Other Hispanic	37.7	45.7	38.0	35.6	**
Mexican American	62.3	54.3	62.0	67.4	**

Tests of Significance = *** *p* < 0.01, ** *p* < 0.05, and * *p* < 0.1. Chi-Square Test for Categorical Variables; Adjusted Wald Test for Continuous Variables.

**Table 2:** Sample characteristics of Hispanic adult respondents within the Vegetables Dietary Pattern included in the National Health and Nutrition Examination Survey, 2013–2018 (n = 2049).

Characteristics		Tertiles			
	Overall	T1	T2	T3	
N	2049	683	683	683	
Age, Mean (In Years)	40.2	37.2	40.1	41.3	**
Sex, %					
Female	51.7	48.3	56.2	49.7	**
Male	48.3	51.7	43.8	50.3	**
Household Annual Income, %					
USD 0–19,999	20.8	30.1	23.8	15.3	***
USD 20,000–34,999	21.4	23.9	20.4	21.2	***
USD 35,000+	57.8	46.0	55.8	63.5	***
Education Level, %					
Less than High School Education	34.6	38.6	33.9	33.7	
High School Diploma	21.9	21.9	22.6	21.5	
Some College +	43.4	39.5	43.5	44.8	
Nativity and Duration of Residence, %					
US Born	49.1	53.4	55.5	43.0	**
Living In the US <10 Years	10.6	11.9	9.7	10.7	**
Living in the US for 10 Years+	40.3	34.7	34.8	46.3	**
Race/Hispanic Origin, %					
Other Hispanic	37.7	44.0	37.5	35.7	
Mexican American	62.3	56.1	62.5	64.3	

Tests of Significance = *** *p* < 0.01 and ** *p* < 0.05. Chi-Square Test for Categorical Variables; Adjusted Wald Test for Continuous Variables.

**Table 3:** Sample characteristics of Hispanic adult respondents within the Plant-Based Dietary Pattern included in the National Health and Nutrition Examination Survey, 2013–2018 (n = 2049).

Characteristics		Tertiles			
	Overall	T1	T2	T3	
N	2049	683	683	683	
Age, Mean (In Years)	40.2	39.9	39.8	41.0	
Sex, %					
Female	51.7	47.4	54.4	54.4	*
Male	48.3	52.6	45.6	45.6	*
Household Annual Income, %					
USD 0–19,999	20.8	21.8	22.8	18.3	
USD 20,000–34,999	21.4	22.2	22.2	19.4	
USD 35,000+	57.8	56.7	55.1	62.3	
Education Level, %					
Less than High School Education	34.6	32.5	40.5	30.7	***
High School Diploma	21.9	23.9	22.8	18.3	***
Some College +	43.4	43.6	36.7	51.0	***
Nativity and Duration of Residence, %					
US Born	49.1	51.9	48.3	46.4	
Living In the US <10 Years	10.6	8.8	13.1	10.0	
Living in the US 10 Years +	40.3	39.3	38.6	43.6	
Race/Hispanic Origin, %					
Other Hispanic	37.7	38.0	34.1	41.5	
Mexican American	62.3	62.0	65.9	58.6	

Tests of Significance = *** *p* < 0.01, * *p* < 0.1. Chi-Square Test for Categorical Variables, Adjusted Wald Test for Continuous Variables.

**Table 4:** Association between dietary patterns and cardiometabolic outcomes for Hispanic adult respondents included in the National Health and Nutrition Examination Survey, 2013–2018.

	Model 1			Model 2			Model 3			Model 4		
	Body Mass Index (n = 2088)			Total Body Fat, % (n = 1255)			Diabetes, HbA1c ≥ 6.5 (n = 2048)			Glycohemoglobin (HbA1c) (n = 2048)		
	βcoef	[95% CI]		βcoef	[95% CI]		OR	[95% CI]		βcoef	[95% CI]	
Dietary Patterns (Tertiles)												
Solid Fats, Cheeses, Refined Carbohydrates												
T1	ref			ref			ref			ref		
T2	1.07	0.14	1.99	0.98	−0.28	2.23	1.04	0.57	1.89	0.02	−0.19	0.23
T3	1.11	−0.04	2.26	0.82	−0.30	1.96	0.98	0.51	1.89	0.09	−0.11	0.28
Vegetables (High in Red and Orange Vegetables, Dark Greens, Tomatoes)												
T1	ref			ref			ref			ref		
T2	−0.04	−1.20	1.12	−0.45	−1.83	0.93	1.89	0.93	3.86	0.03	−0.14	0.20
T3	−0.93	−1.92	0.06	−1.57	−2.74	−0.39	1.42	0.67	2.97	−0.01	−0.19	0.18
Plant Based (High in Soy, Nuts, Seeds, and Fruit)												
T1	ref			ref			ref			ref		
T2	0.18	−0.72	1.09	0.19	−0.75	1.14	1.25	0.85	1.83	0.09	−0.12	0.14
T3	−0.17	−1.51	1.17	−0.29	−1.49	0.89	0.88	0.56	1.38	−0.06	−0.23	0.11

Covariates for all models included age, sex, education level, income level, and caloric intake. For models 3 and 4, additional covariates included whether participants were on a special diet tailored for diabetes, were currently taking insulin, and were currently taking medication to control blood glucose levels.

**Table 5:** Association between Dietary Patterns and Cardiometabolic Outcomes for Hispanic adult respondents included in the National Health and Nutrition Examination Survey, 2013–2018.

	Model 5			Model 6			Model 7			Model 8		
	History of Coronary Heart Disease (n = 1953)			History of Myocardial Infarction (n = 1955)			Average Systolic Blood Pressure (mmhg) (n = 2080)			Average Diastolic Blood Pressure (mmhg) (n = 2080)		
	OR	[95% CI]		OR	[95% CI]		βcoef	[95% CI]		βcoef	[95% CI]	
Dietary Patterns (Tertiles)												
Solid Fats, Cheeses, Refined Carbohydrates												
T1	ref			ref			ref			ref		
T2	2.28	0.85	6.16	0.58	0.19	1.73	−0.55	−2.96	1.86	−0.86	−2.86	1.14
T3	2.26	0.92	5.57	0.76	0.33	1.74	−2.47	−4.89	−0.59	−1.29	−3.68	1.08
Vegetables (High content of Red and Orange Vegetables, Dark Greens, Tomatoes)												
T1	ref			ref			ref			ref		
T2	3.05	0.98	9.53	0.82	0.22	3.06	−1.13	−3.32	1.07	−0.73	−2.78	1.32
T3	2.96	0.91	9.60	0.88	0.32	2.50	−0.79	−2.47	0.88	−0.48	−2.42	1.47
Plant Based (High content of Soy, Nuts, Seeds, and Fruit)												
T1	ref			ref			ref			ref		
T2	1.1	0.56	2.17	1.03	0.54	2.01	−0.20	−2.42	2.02	−0.05	−1.44	1.35
T3	1.16	0.51	2.63	1.47	0.67	3.25	−1.48	−3.82	0.85	−0.26	−19.97	1.45

Covariates for all models included age, sex, education level, income level, caloric intake, and smoking status.

**Table 6:** Association between dietary patterns and cardiometabolic outcomes for Hispanic adult respondents from the National Health and Nutrition Examination Survey, 2013–2018.

	Model 9			Model 10			Model 11		
	HDL Cholesterol (mg/dL) (n = 2037)			Fasted LDL Cholesterol (mg/dL) (n = 894)			Fasted Triglycerides (mg/dL) (n = 908)		
	βcoef	[95% CI]		βcoef	[95% CI]		βcoef	[95% CI]	
Dietary Patterns (Tertiles)									
Solid Fats, Cheeses, Refined Carbohydrates									
T1	ref			ref			ref		
T2	−1.58	−4.11	0.96	5.98	−1.67	13.63	5.88	−11.45	23.22
T3	−4.53	−7.03	−2.03	5.47	−4.55	15.48	−15.4	−44.08	13.25
Vegetables (High content of Red and Orange Vegetables, Dark Greens, Tomatoes)									
T1	ref			ref			ref		
T2	−2.62	−4.79	−0.47	−0.37	−8.87	8.12	16.84	−11.59	45.28
T3	0.51	−1.45	2.47	0.64	−9.78	11.06	−2.84	−23.63	17.93
Plant Based (High content of Soy, Nuts, Seeds, and Fruit)									
T1	ref			ref			ref		
T2	−1.29	−3.46	0.88	−0.26	−5.57	5.04	−15.8	−32.14	0.64
T3	1.22	−0.69	3.14	2.44	−3.73	8.62	−12.4	−32.39	7.60

Covariates for all models included age, sex, education level, income level, and caloric intake. For model 9, an additional covariate of physical activity level (METs per minute per week) was also included. For model 11, an additional covariate of glycohemoglobin level was included.

## Data Availability

Data from the NHANES can be found at https://www.cdc.gov/nchs/nhanes/index.htm (accessed on 6 September 2023). The FPED is available at https://www.ars.usda.gov/northeast-area/beltsville-md-bhnrc/beltsville-human-nutrition-research-center/food-surveys-research-group/docs/fped-overview/ (accessed on 6 September 2023).

## Supplementary Materials

The following supporting information can be downloaded at https://www.mdpi.com/article/10.3390/nu15214641/s1. A supplemental table is included that is meant to be published alongside the manuscript. Table S1. Factor Loadings for Dietary Patterns, Hispanic Adult Respondents, National Health and Nutrition Examination Survey, 2013–2018 (n = 2049)
