# Supplementary material for: Dietary Patterns and Their Association with Cardiometabolic Biomarkers and Outcomes among Hispanic Adults: A Cross-Sectional Study from the National Health and Nutrition Examination Survey (2013–2018)
Source: Nutrients. 2023 Nov 1;15(21):4641. doi: 10.3390/nu15214641 (PMC10647485; doi:10.3390/nu15214641)
Supplement: Supplementary file 1 [file nutrients-15-04641-s001.zip › nutrients-2662297-supplementary.pdf]

nutrients MDPI

Table S1. Factor Loadings for Dietary Patterns, Hispanic Adult Respondents, National Health and Nutrition Examination Survey, 2013–2018 (n = 2049).

Food Groups (n = 28)	Factor 1	Factor 2	Factor 3
	**Solids Fats, Cheese, & Refined Carbohydrates**	**Vegetables**	**Plant-Based**
Solid Fats	**0.84**	0.01	0.01
Refined Grains	**0.81**	0.04	0.01
Cheeses	**0.78**	0.11	−0.04
Added Sugars	**0.52**	−0.19	0.02
Added Oils	**0.39**	0.19	0.28
Vegetables, Other (Not Already Listed)	0.19	**0.73**	0.02
Red and Orange Vegetables (exlclud. Tomatoes/Tomato Products)	−0.12	**0.58**	**0.35**
Tomato and Tomato Products (includ. Ketchup)	**0.47**	**0.49**	−0.12
Dark Green Vegetables	−0.09	**0.40**	0.02
Soy Products	−0.01	0.03	**0.79**
Nuts and Seeds	0.02	0.00	**0.61**
Whole Fruits (exclud. Melons, Citrus, Berries)	−0.06	0.28	**0.45**
Whole Grains	−0.12	0.12	0.01
White Potatoes	0.03	−0.06	−0.02
Poultry	−0.02	0.14	−0.04
Beef, Veal, Pork, Lamb, and Game Meat	0.22	0.21	−0.03
Seafood Low in Omega-3 Fatty Acids	−0.05	0.05	0.08
Seafood High in Omega-3 Fatty Acids	−0.05	0.00	0.06
Eggs	0.18	0.07	0.05
Cured Meats	0.18	−0.13	−0.03
Fruit Juice	0.02	0.08	−0.24
Other Starchy Vegetables (exclud. White Potatoes)	−0.08	0.18	0.27
Alcohol	0.03	0.10	−0.15
Beans and Peas Computed as Vegetables	0.21	0.02	−0.04
Yogurts	−0.02	0.03	0.11
Whole Fruits (Melons, Citrus, Berries only)	−0.06	0.27	−0.03
Organ Meat	−0.03	0.04	−0.03
Milk	0.32	−0.20	0.09
Dietary Variance Explained, %	11.86	8.72	5.20
Eigenvalue	3.32	2.44	1.45
